# EGFR Tyrosine Kinase Inhibitors Activate Autophagy as a Cytoprotective Response in Human Lung Cancer Cells

**DOI:** 10.1371/journal.pone.0018691

**Published:** 2011-06-02

**Authors:** Weidong Han, Hongming Pan, Yan Chen, Jie Sun, Yanshan Wang, Jing Li, Weiting Ge, Lifeng Feng, Xiaoying Lin, Xiaojia Wang, Xian Wang, Hongchuan Jin

**Affiliations:** 1 Department of Medical Oncology, Biomedical Research Center, Sir Run Run Shaw Hospital, School of Medicine, Zhejiang University, Hangzhou, Zhejiang, China; 2 Laboratory of Cancer Epigenetics, Biomedical Research Center, Sir Run Run Shaw Hospital, School of Medicine, Zhejiang University, Hangzhou, Zhejiang, China; 3 Zhejiang Cancer Hospital, Zhejiang Chinese Medical University, Hangzhou, Zhejiang, China; 4 Cancer Institute, The Second Affiliated Hospital, College of Medicine, Zhejiang University, Hangzhou, Zhejiang, China; University of Pennsylvania, United States of America

## Abstract

Epidermal growth factor receptor tyrosine kinase inhibitors gefitinib and erlotinib have been widely used in patients with non-small-cell lung cancer. Unfortunately, the efficacy of EGFR-TKIs is limited because of natural and acquired resistance. As a novel cytoprotective mechanism for tumor cell to survive under unfavorable conditions, autophagy has been proposed to play a role in drug resistance of tumor cells. Whether autophagy can be activated by gefitinib or erlotinib and thereby impair the sensitivity of targeted therapy to lung cancer cells remains unknown. Here, we first report that gefitinib or erlotinib can induce a high level of autophagy, which was accompanied by the inhibition of the PI3K/Akt/mTOR signaling pathway. Moreover, cytotoxicity induced by gefitinib or erlotinib was greatly enhanced after autophagy inhibition by the pharmacological inhibitor chloroquine (CQ) and siRNAs targeting ATG5 and ATG7, the most important components for the formation of autophagosome. Interestingly, EGFR-TKIs can still induce cell autophagy even after EGFR expression was reduced by EGFR specific siRNAs. In conclusion, we found that autophagy can be activated by EGFR-TKIs in lung cancer cells and inhibition of autophagy augmented the growth inhibitory effect of EGFR-TKIs. Autophagy inhibition thus represents a promising approach to improve the efficacy of EGFR-TKIs in the treatment of patients with advanced non-small-cell lung cancer.

## Introduction

Lung cancer is the leading cause of cancer deaths worldwide [1]. Chemotherapy is still not effective enough for patients with advanced non–small-cell lung cancer (NSCLC) and the response rate is only 20% to 35% with a median survival of 10 to 12 months [2], [3]. By targeting molecules critical to cancer development, targeted therapy alone or in combination with other treatments was recently recognized as a promising strategy to conquer cancers including NSCLC [4].

As one of receptor tyrosine kinases (RTKs) important to cancer cell growth, proliferation, invasion, and metastasis, epidermal growth factor receptor (EGFR) was frequently deregulated in NSCLCs [5]. EGFR over-expression was observed in about 62% of squamous cell and adenocarcinoma subtypes [6]. EGF can induce the activation of three signaling pathways important to the initiation and progression of cancers, Ras/MAPK, PI3K/Akt, and JAK/STATs [7]. As a result, EGFR became one of the molecules for the development of targeted therapy to NSCLC. By inhibiting the tyrosine kinase activity of EGFR, two tyrosine kinase inhibitors (TKIs) named gefitinib (Iressa, AstraZeneca) and erlotinib (Tarceva, Genentech) have been developed for the treatment of NSCLC. Gefitinib and erlotinib can inhibit tumor growth both in vitro and in vivo. Clinically, both EGFR-TKIs showed good tolerability and antitumor activity in NSCLC patients with disease progressing after first line platinum-based chemotherapy [5], [8], [9]. However, the efficacy of EGFR-TKIs is significantly decreased by natural and acquired resistance. The mechanism is still largely unknown although EGFR mutations have been proposed to be one of mechanisms to influence the sensitivity of EGFR to these inhibitors [10], [11].

Macroautophagy (hereafter referred to as autophagy) is a self-proteolysis process in eukaryotic cells that results in the breakdown of intracellular material within macroautophagosome or lysosomes [12], [13]. Under cellular stress conditions such as nutrient-deficient environment, autophagy is rapidly activated to provide an alternative source of energy and thus enable cells to survive [14]. Autophagy was upregulated during the later stage of tumor growth because induction of autophagy allows tumor cells to survive in microenvironments lack of nutrient and oxygen [15]. Through promoting the survival of tumor cells under unfavorable conditions, autophagy was proposed as an alternative mechanism of drug resistance. For example, autophagy contributes to the resistance of breast cancer cells to bortezomib treatment [16]. Inhibition of autophagy could sensitize tumor cells to many cytotoxic drugs or reverse the resistance to chemotherapeutic drugs, representing a promising strategy to improve the efficacy of cancer treatment [17].

Signaling pathways downstream of EGFR and other RTKs such as PI3K/Akt pathway are involved in the regulation of autophagy, indicating a potential link between RTK inhibition and autophagy. Another TKI named as imatinib indeed can activate autophagy in respective of cell types [18], [19]. In addition, blockade of macroautophagosome formation enhances the efficacy of anti-HER2 monoclonal antibody trastuzumab (Tzb) [20]. However, whether autophagy is associated with gefitinib and erlotinib treatment in lung cancer cells remains unknown. In the current study, we first demonstrate that gefitinib or erlotinib activated autophagy in lung cancer cells and blockage of autophagy enhanced the effect of gefitinib or erlotinib.

## Materials and Methods

### Reagents and antibodies

The chemicals used were gefitinib (J&K chemical Ltd., G304000), erlotinib (J&K chemical Ltd., E625000) and chloroquine (CQ) (J&K chemical Ltd., 147236). The Primary antibodies were antibodies against microtubule-associated protein 1 light chain 3 (LC3) (Cell Signaling Technology, #2775), ATG5 (Cell Signaling Technology, #2630), ATG7 (Cell Signaling Technology, #2631), phospho-mTOR (S2448) (Cell Signaling Technology, #2971), total mTOR (Cell Signaling Technology, #2983), phospho-P70S6K (T389) (Cell Signaling Technology, #9234), phospho-AKT (S473) (Cell Signaling Technology, #4051), total AKT (Cell Signaling Technology, #9272), GAPDH (Cell Signaling Technology, #3683). The secondary antibodies were HRP conjugated anti-rabbit (Santa Cruz Biotechnology, sc-2357) and anti-mouse IgG (Santa Cruz Biotechnology, sc-2371).

### Cell cultures

The lung cancer cell lines (A549, NCI-H1299, NCI-H292, NCI-H1650 and SK-MES-1) were bought from cell bank (Chinese Academy of Sciences). Monolayer culture of cancer cells was maintained in RPMI 1640 supplemented with 10% (v/v) FBS, and antibiotics. Stock solution of gefitinib or erlotinib was prepared in dimethyl-sulphoxide (DMSO) (Sigma, D4540), and diluted with medium before use. Final concentration of DMSO was <0.1%.

### Cytotoxicity assay

The cytotoxicity of chemicals against lung cancer cell lines was determined by MTT assay. Cells were seeded into 96-well plates (cultured overnight for adherent cells) and treated with chemicals with different concentrations. After 48-h incubation, 20 µl MTT (5 mg/ml) was added into each well for 4-h incubation. After that, the supernatant was removed and 150 µl DMSO was added into each well in order to solubilize the blue-purple crystals of formazan. The absorbance was then measured using a model ELX800 Micro Plate Reader (Bio-Tek Instruments, Inc.) at 570 nm.

### Immunofluorescent confocal laser microscopy For LC3 and lysosome co-location

Lysosome was firstly labeled by incubation with Lyso Tracker (Invitrogen, L7528), a lysosome reporter dye, for 90 min at 37°C. Cells were collected, fixed and permeabilized with 1% CHAPS buffer (150 mM NaCl, 10 mM HEPES, 1.0% CHAPS) at room temperature for 10 min, incubated with anti-LC3 for 2 h at room temperature, and washed with PBS, incubated for another 45 min with FITC-conjugated goat anti-rabbit IgG (Beyotime, A0562). Then, cell nuclei were stained by DAPI (Sigma, D9564). Samples were examined under a Zeiss LSM 710 confocal microscope system (Carl Zeiss, Germany). Image was processed with ZEN LE software.

### Electron microscopy

Treated cells were washed and fixed for 30 min in 2.5% glutaraldehyde. The samples were treated with 1.5% osmium tetroxide, dehydrated with acetone and embedded in Durcupan resin. Thin sections were poststained with lead citrate and examined in the TECNAI 10 electron microscope (Philips, Holland) at 60 kV.

### Western blot analysis

Western blotting was carried out as previously reported [21]. The protein was applied to a proper concentration of SDS-polyacrylamide gel, transferred to a PVDF membrane, and then detected by the proper primary and secondary antibodies before visualization with a chemiluminescence kit. Visualization was done with Image Quant LAS-4000 (Fujifilm, Tokyo, Japan) using image Multi-Gauge Software (Fujifilm, Tokyo, Japan).

### RNA interference

Cells were transfected with either nonspecific siRNA (Qiagen, 1027280), ATG5 siRNA (Qiagen, SI02655310), ATG7 siRNA (Qiagen, SI02655373) or EGFR siRNA (Cell Signaling Technology, #6481) (siRNA final concentration, 100 nmol/L) via LipofectAMINE RNAi max (Invitrogen, 13778150) according to the manufacturer's instructions. Cells were then incubated for 48 h prior to Western blot or MTT assay.

### Real-time PCR

Total RNA was isolated using the Trizol method and cDNA was synthesized from mRNA with the PrimeScipt MMLV RT reagent Kit (TakaRa, #6110). Real-time quantitative PCR reaction was carried out using SYBR green as the fluorescent reporter (Bio-Rad, 170-8882). QPCR experiments were done on an ABI-7500 and GAPDH was served as an endogenous control. Each sample was normalized on the basis of its GAPDH content. A total of 40 cycles (5-s denaturation at 95°C, 34-s annealingt/extension at 55°C) were run for each primer. Primer sequences were as follows: for ATG5, TTC AAT CAG GTT TGG TGG AGG C (sense) and ATG GCA GTG GAG GAA AGC AGA G (antisense); for ATG7, ATG CCT GGG CAT CCA GTG AAC TTC (sense) and CAT CAT TGC AGA AGT AGC AGC CA (antisense); for GAPDH, GGA GTC AAC GGA TTT GGT (sense) and GTG ATG GGA TTT CCA TTG AT (antisense).

### Statistical analyses

Unless otherwise stated, data were expressed as the mean ± SD, and analyzed by Student's t test.

## Results

### EGFR-TKIs induce autophagy in lung cancer cells

**Figure 1:**
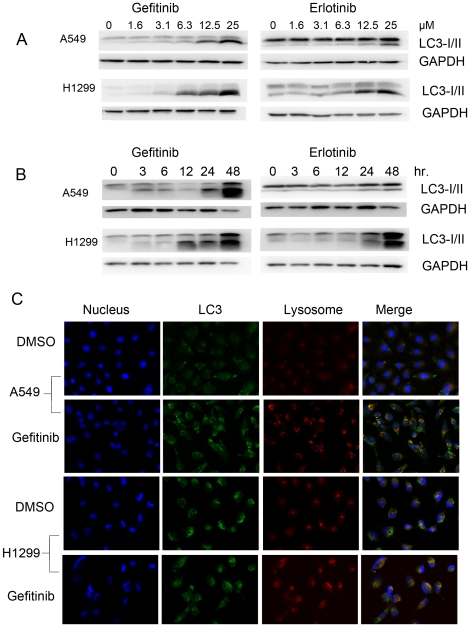
EGFR-TKIs dose- and time-dependently induced the formation of LC3-II, and formation of autolysosomes in lung cancer cells. **A** and **B**, lung cancer cells were incubated with varying concentrations of gefitinib or erlotinib for 24 hours or incubated with 25 µM gefitinib or erlotinib for appropriate intervals. The switch of LC3-I to LC3-II was detected by immunoblotting. **C**, A549 or NCI-H1299 cells were treated with DMSO or 25 µM gefitinib for 24 h. Cells were labeled with fluorescence and imaged by confocal microscope. Red, lyso tracker-labeled lysosomes; Green, FITC-labeled LC3; Blue, DAPI-labeled nucleus. The overlay was shown in the right column. The orange-stained cells indicated LC3 co-located with lysosomes.

**Figure 2:**
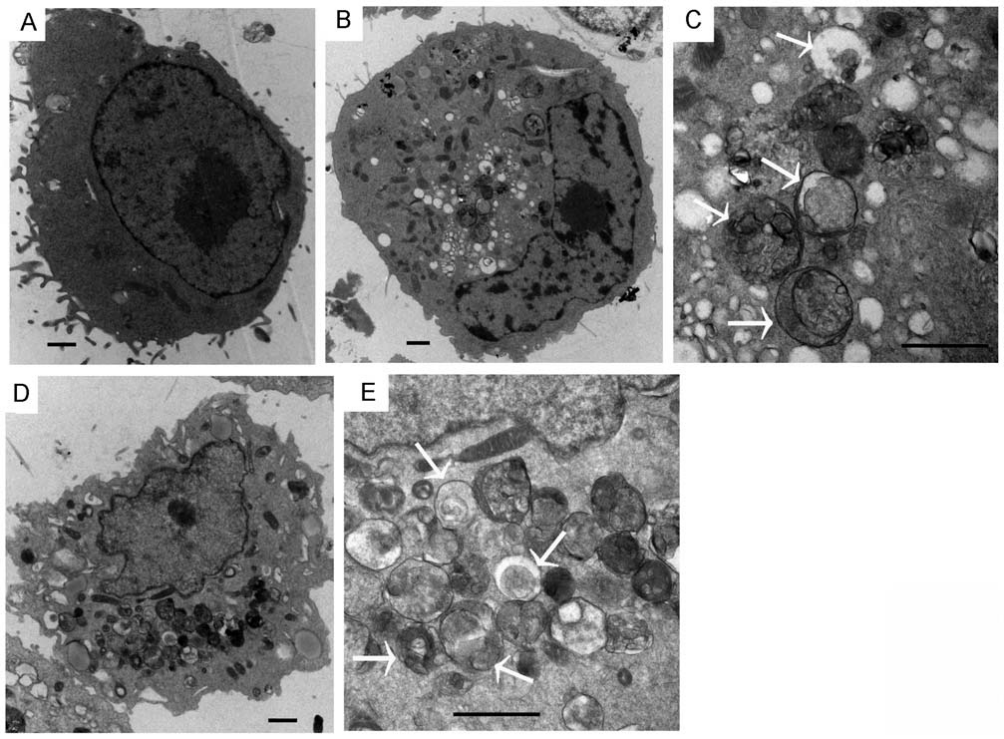
TEM depicting ultrastructures of autophagosome in A549 cells treated EGFR-TKIs. A549 cells were treated with 25 µM gefitinib for 24 h (**A**, DMSO; **B**, Gefitinb, low power; **C**, Gefitinb, high power). **D** (low resolution) and **E** (high resolution), AGS cells were treated with 25 µM erlotinib for 24 h (**D**, erlotinib, low power; **E**, erlotinib, high power). Numerous autophagical vacuoles with typical double-layer membrane containing organelle remnants were highlighted by arrows. Bar = 1 µm.

To evaluate the activation of autophagy by EGFR-TKIs, the conversion of LC3-I into LC3-II before and after TKIs treatment was determined by western blotting analysis. In A549 and NCI-H1299 cells, both gefitinib and erlotinib can induce the switch of LC3-I to LC3-II in the dose and time dependent manner, indicating that autophagy might be activated by both TKIs (Figure 1A and B). To further confirm it, the compartmentalization of LC3 in cells before and after TKIs treatment was monitored by indirect immunofluorescence staining. Indeed, TKIs induced the colocalization of LC3-II with lysosome, demonstrating the formation of autolysosome (Figure 1C and Figure S1). Ultrastructural analysis by electron microscopy further confirmed that numerous large autophagic vacuoles with typical double-layer membrane containing organelle remnants presented in gefitinib-treated A549 cells rather than untreated cells (Figure 2A, B and C). Similar results were obtained with erlotinib (Figure 2D and E).

**Figure 3:**
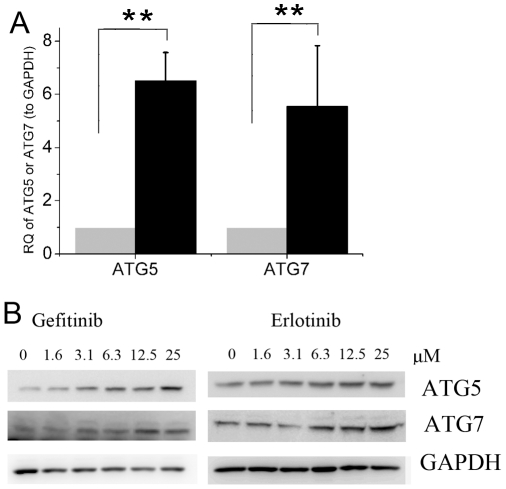
Effects of EGFR-TKIs on the expression of ATG5 and ATG7. **A**, A549 cells were treated with 25 µM gefitinib for 6 h and the mRNA level of ATG5/7 was measured by Real time RT-PCR as described in M&M. RQ, relative quantity. black, gefitinib-treated cells; gray, control cells. **B**, Immunoblotting for Atg5 or Atg7 using lysates from A549 treated with varying concentrations of gefitinib or erlotinib for 24 hr. Data were expressed as the mean ± SD, and analyzed by Student's t test. ** P<0.01.

We next examined the effects of EGFR-TKIs on the expression of ATG5 and ATG7, two critical components in regulating the formation of autophagosomes [22]. Both EGFR-TKIs increased ATG5 and ATG7 at the mRNA or protein levels (Figure 3), confirming the induction of autophagy by EGFR-TKIs.

### Inhibition of Akt/mTOR/p70S6K signaling pathway by EGFR-TKIs

**Figure 4:**
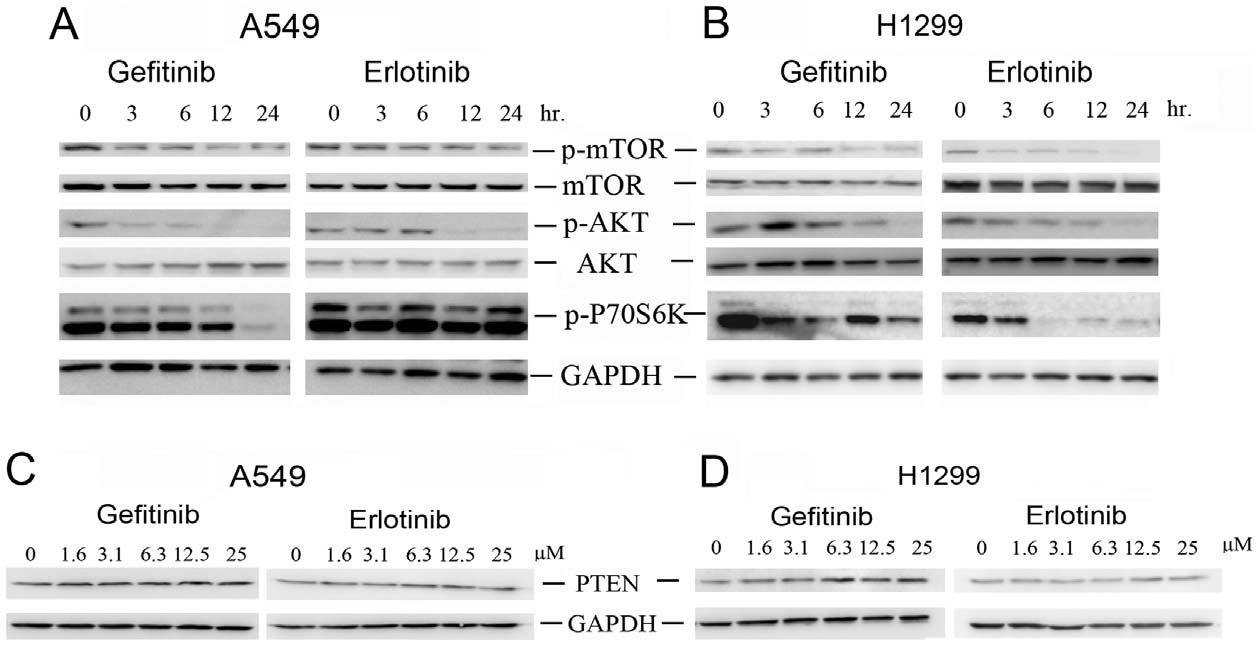
EGFR-TKIs induced autophagy by inhibiting the Akt/mTOR/p70S6K signaling pathway in A549 and H1299 cells. The phosphorylation of mTOR, Akt and p70s6k in A549 (**A**) or NCI-H1299 (**B**) cells treated with gefitinib or erlotinib were determined by western blotting. The expression of PTEN in A549 (**C**) or NCI-H1299 (**D**) cells treated with varying concentrations of gefitinib or erlotinib were detected by western blotting.

Previous studies have shown that PI3K/Akt/mTOR/p70S6K axis plays an important role in the inhibition of autophagy [23], [24], we therefore investigate the effect of gefitinib and erlotinib on this autophagy-suppressive signaling pathway. Indeed, phosphorylation of AKT, mTOR and p70S6K in both A549 and H1299 cells were significantly reduced by gefitinib and erlotinib in a time-dependent manner (Figure 4A and B). However, no changes of PTEN expression were detected after TKIs treatment (Figure 4C and D).

### *Blockage of autophagy enhance EGFR-TKIs-induced cell death*

**Figure 5:**
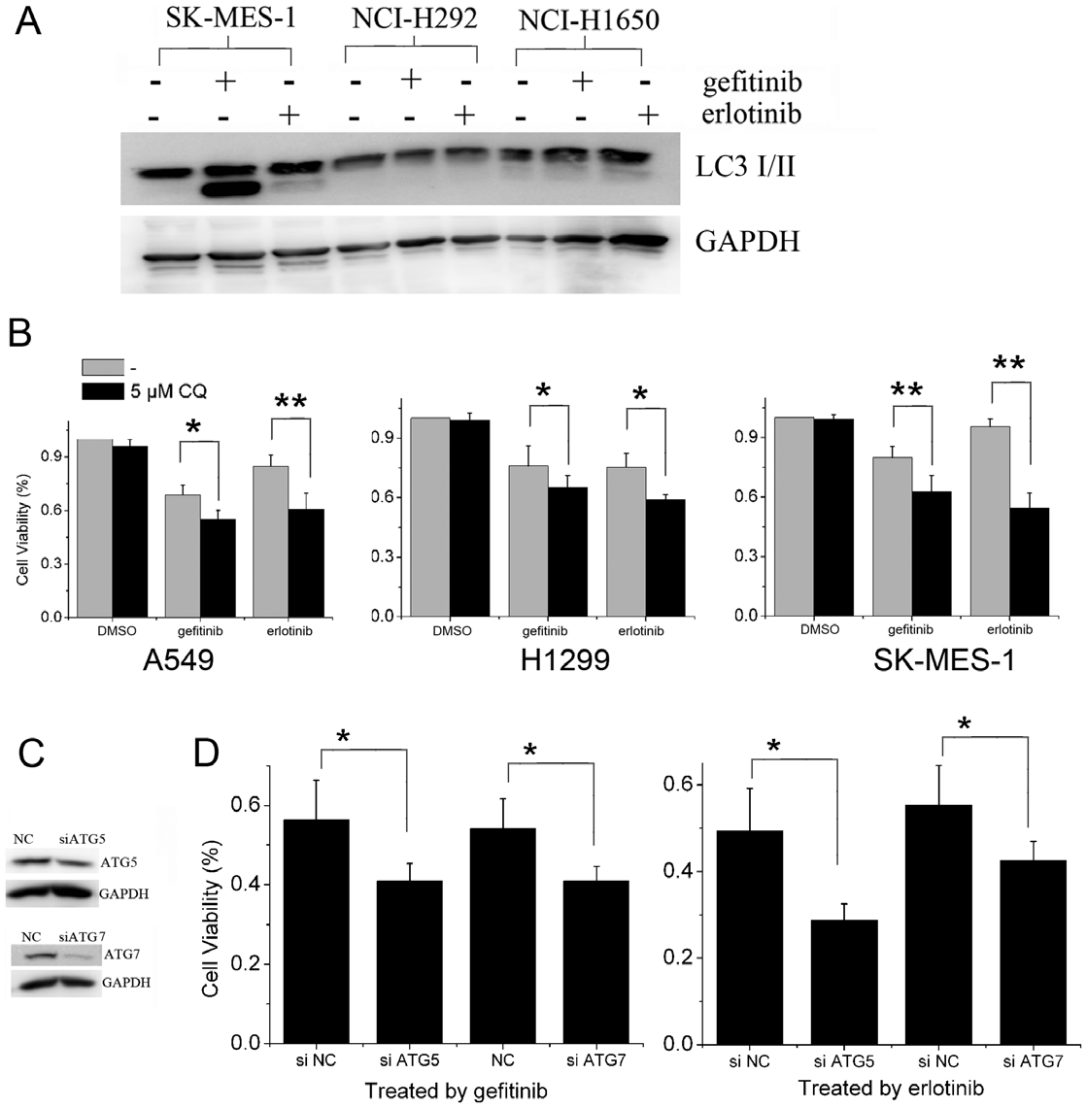
Inhibition of autophagy enhances the cytotoxity of EGFR-TKIs in lung cancer cells. **A**, Autophagy was induced by EGFR-TKIs in resistant but not sensitive lung cancer cells. LC3 expression in lung cancer cells incubated with 25 µM gefitinib or erlotinib for 24 h were analyzed by immunoblotting. **B**, The viability of cancer cells treated with 12.5 µM gefitinib or erlotinib for 48 h with or without 5 µM CQ were measured by MTT assay. **C**, The expression of ATG5 and ATG7 in A549 cells transiently transfected with negative control siRNA, Atg7 or Atg5 siRNA were determined by immunoblotting. **D**, Cells transiently transfected with negative control siRNA, Atg7 or Atg5 siRNA were treated with 12.5 µM gefitinib or 25 µM erlotinib for 48 h. The effect of EGFR-TKIs on cancer cells with or without ATG5 or ATG7 knockdown were analyzed by MTT assay. NC, Control siRNA. Data were expressed as the mean ± SD, and analyzed by Student's t test. * P<0.05, ** P<0.01.

Many studies have demonstrated that autophagy may serve as a protective response preventing tumor cells from therapy-induced cell death [17], [20], [25], [26], [27], [28], [29], [30]. In lung cancer cell line NCI-H292 and NCI-H1650 that are relatively sensitive to gefitinib and erlotinib, no autophagy was detected after gefitinib or erlotinib treatment (Figure 5A). However, autophagy was detected in EGFR-TKIs treated cancer cells that are relatively resistant to EGFR-TKIs, such as A549, NCI-H1299 and SK-MES-1 cells (Figure 1 and 5A). These data indicated that autophagy may impair the sensitivity of cancer cells to EGFR-TKIs. To test this consumption, we compared the growth inhibitory effect of gefitinib or erlotinib on cancer cells before and after pharmacological and genetic inhibition of autophagy. As shown in Figure 5B, CQ which can impair the function of lysosomes and inhibit autophagy at late stage significantly augmented growth inhibition induced by gefitinib or erlotinib in A549, NCI-H1299 and SK-MES-1 that are relatively resistant to EGFR-TKIs. However, it could not inhibit lung cancer cell growth by its own. (Figure 5B). Similarly, growth inhibition induced by gefitinib or erlotinib in A549 cells was enhanced after autophagy was inhibited by the knockdown of ATG5 or ATG7 (Figure 5C and D), two essential components for the formation of autophagosome, confirming that autophagy protected tumor cells from EGFR-TKIs induced cell death.

### EGFR-TKIs induce autophagy in cancer cells with EGFR-knockdown

**Figure 6:**
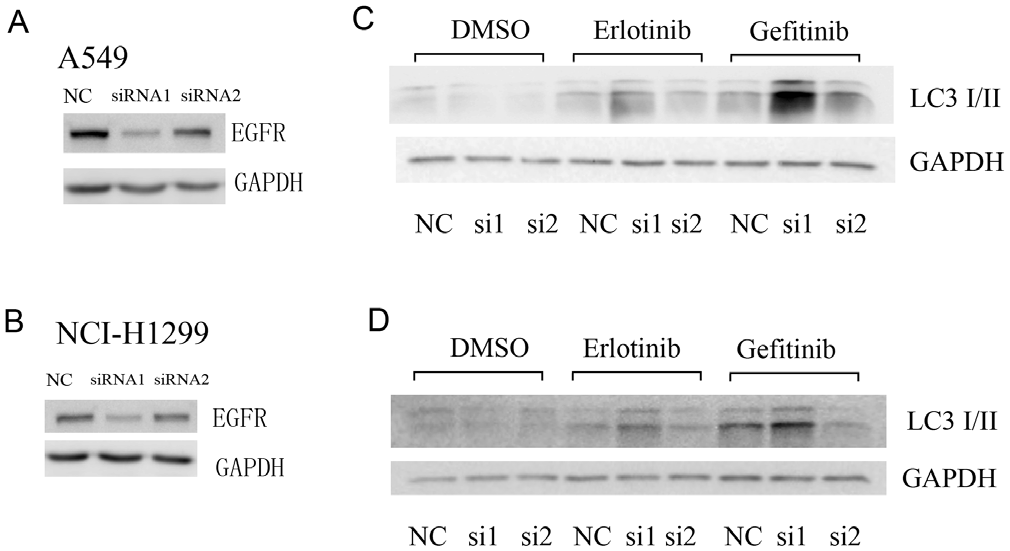
EGFR-TKIs induced autophagy in EGFR-knockdown cells. EGFR expression in A549 (**A**) or H1299 (**B**) cells transfected with control siRNA, EGFR siRNA1 or siRNA2 were determined by western blotting. LC3 expression in EGFR-TKIs treated A549 (**C**) or H1299 (**D**) cells with or without EGFR knockdown were determined by western blotting.

Next, we would like to know the role of EGFR in the gefitinib or erlotinib induced autophagy. Two specific siRNAs were used to knockdown EGFR expression. SiRNA 1 could reduce EGFR expression greatly while siRNA 2 which had moderate effect on EGFR expression (Figure 6A and B). Interestingly, autophagy activated by either gefitinib or erlotinib was not inhibited but augmented after EGFR expression was dramatically reduced by siRNA 1 (Figure 6C and D). In contrast, siRNA 2 hardly had any influence on autophagy induced by both EGFR-TKIs (Figure 6C and D).

## Discussion

Autophagy was designated as programmed cell death type II, whereas apoptosis is well-known as programmed cell death type I. However, recently autophagy was found to promote cellular survival under unfavorable conditions such as deprivation of amino acids or ATPs, revealing a new role of autophagy in cancer development.

Autophagy is morphologically characterized by the appearance of “double-membrane” vacuoles (autophagosomes) in the cytoplasm. In addition, the mammalian homologue of the yeast protein Apg8p, (also named LC3), is found to be a specific biochemical marker for autophagy. Newly synthesized LC3 termed LC3-1 is evenly distributed throughout the cytoplasm. Upon induction of autophagy, some LC3-I is converted into LC3-II, which is tightly bound to the autophagosomal membranes forming ring-shaped structures in the cytosol. We confirmed biochemically and morphologically that autophagy can be activated by gefitinib or erlotinib, two well-used EGFR-TKIs, in two independent lung cancer cell lines. Interestingly, a very recent paper reported that autophagy can be induced by anti-EGFR antibodies [31]. Both EGFR antibodies and EGFR-TKIs are widely used to treat cancer patient by inhibition of EGFR, revealing a new link between EGFR inhibition and autophagy activation.

Autophagy can be activated as the cellular response to cancer therapy. A number of cancer therapeutics including DNA-damaging chemotherapeutics, endocrine therapies (e.g. tamoxifen) and radiation therapy have been found to induce autophagy in vitro and in vivo [32], [33], [34]. Recently, it was found that autophagy can be activated and protected tumor cells from targeted therapies, such as the imatinib mesylate in philadelphia chromosome–positive cells [18], trastuzumab in breast cancer [20], Src family kinase inhibitors in prostate cancer [35], proteasome inhibitors in prostate cancer [16]. Consistently, we found that autophagy can be activated by gefitinib and erlotinib in lung cancer and promote cellular survival in the target therapy using EGFR-TKIs. Blockage of autophagy by pharmacological or genetic approaches greatly enhanced the growth inhibitory effect of gefitinib or erlotinib. Thus, inhibition of autophagy has the potential to improve the clinical efficacy of EGFR-TKIs for cancer treatment.

As a sensor of amino acids and ATP, mTOR negatively regulates autophagy. Indeed, we found that both TKIs can inhibit the activation of mTOR as well its upstream regulator, PI3K/Akt. Another signaling pathway important to autophagy is Raf/MAPK pathway [23]. However, we failed to find a clear correlation between Raf/MAPK pathway and autophagy in cell lines we used. This is probably due to oncogenic mutations predominantly occurred in Ras/MAPK pathways. For instance, k-Ras gene was known to be mutated in A549 cells. Clinically, patients with k-Ras mutations failed to response to TKIs treatment. It would be interesting to know whether patients with k-Ras mutations could benefit from the combination of TKIs and inhibitors of autophagy.

Interestingly, our results indicated that gefitinib or erlotinib induced autophagy might be EGFR independent. As shown in Figure 6, both EGFR-TKIs could induce autophagy even EGFR expression was greatly reduced. Although gefitinib and erlotinib were developed as the specific inhibitors targeted to the kinase domain of EGFR, however, recent results indicted that these two inhibitors can have other targets, such as non-receptor tyrosine kinases that acts also upstream of PI3K/Akt/mTOR pathway [36]. Consistently, large scale clinical trials confirmed that measurement of EGFR expression by immunohistochemistry was not useful for picking up patients to be benefited from gefitinib therapy. Certainly, we still could not completely exclude the relevance of EGFR in gefitinib or erlotinib induced autophagy since certain amount of EGFR was still detected after knockdown by EGFR siRNAs. Indeed, EGFR-TKIs activated autophagy was much more enhanced by siRNA 1 which displayed better knockdown efficiency than siRNA 2 (Figure 6B). Hence, we need further investigations to clarify the role of EGFR in the gefitinib or erlotinib-induced autophagy. Nevertheless, EGFR-TKIs and EGFR siRNA had a synergetic effect on the induction of autophagy, which could be specifically inhibited to increase the clinical efficacy of targeted therapy.

In summary, our work reinforced the notion that cancer cells can survive in a stressful environment, following inhibition of critical oncogenic pathways, by inducing autophagy. These tumor cells are primed to resume proliferation once drug concentration drops after drug withdrawal due to toxicity or mutations develop to confer drug resistance. Thus, in combination of therapeutic strategies that aim to inhibit autophagy in patients treated with conventional chemotherapy or novel targeted therapy with EGFR-TKIs represents a promising approach with higher efficacy for cancer patients.

## Supporting Information

Figure S1 **Quantification of autophagy in lung cancer cells treated by gefitinib.** The percentage of cells with orange signals was calculated by counting cells in 3–4 fields under microscope. The data were represented as the mean ± SD.
(TIF)
