# Supplementary figures and images for: EGFR Tyrosine Kinase Inhibitors Activate Autophagy as a Cytoprotective Response in Human Lung Cancer Cells
Source: PLoS One. 2011 Jun 2;6(6):e18691. doi: 10.1371/journal.pone.0018691 (PMC3107207; doi:10.1371/journal.pone.0018691)

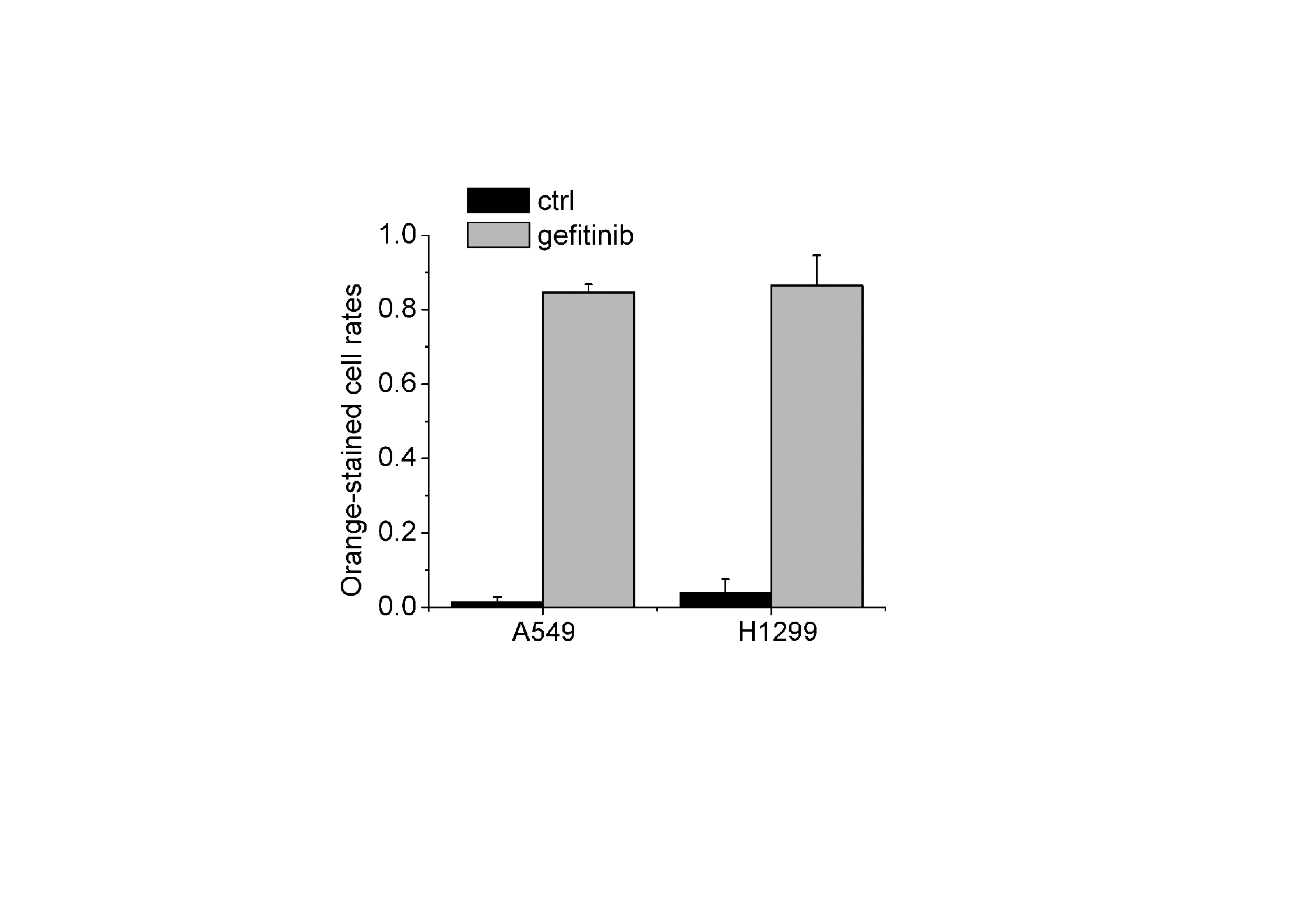

Supplement: Figure S1 — Quantification of autophagy in lung cancer cells treated by gefitinib. The percentage of cells with orange signals was calculated by counting cells in 3–4 fields under microscope. The data were represented as the mean ± SD. (TIF) [file pone.0018691.s001.tif]
